# Case Report: Avelumab and ruxolitinib to manage polycythemia vera and secondary metastatic Merkel cell carcinoma: a possible successful combination

**DOI:** 10.3389/fonc.2023.1212638

**Published:** 2023-06-07

**Authors:** Chiara Masucci, Mauro Passucci, Emilia Scalzulli, Ida Carmosino, Marcello Capriata, Alessandro Costa, Claudia Ielo, Maurizio Martelli, Massimo Breccia

**Affiliations:** ^1^ Department of Translational and Precision Medicine, Policlinico Umberto I, La Sapienza University of Rome, Rome, Italy; ^2^ Hematology Unit, Businco Hospital, Azienda di Rilievo Nazionale ed Alta Specializzazione (ARNAS) G. Brotzu, Cagliari, Italy

**Keywords:** avelumab, Merkel cell carcinoma, ruxolitinib, polycytemia vera, second malignancies

## Abstract

We describe a case of second primary malignancy in a 65-year-old patient affected by polycythemia vera treated with the JAK 1/2 inhibitor ruxolitinib. The latter is recognized as a risk factor for the onset of non-melanoma skin cancers in many retrospective and perspective studies, but the concomitant use of ruxolitinib with new immunotherapies is very rarely reported, and the safety of this association is still not clear. In our case, ruxolitinib combined with the anti-PD-L1 avelumab demonstrated both safety and efficacy for hematological disease control and underlying carcinoma remission.

## Introduction

Second primary malignancies (SPM) are not uncommon in myeloproliferative neoplasms (MPNs), as reported in retrospective studies (1). Ruxolitinib (RUX) is the first in the class JAK1/2 inhibitor approved for symptomatic myelofibrosis (MF) and in second-line therapy in patients with polycythemia vera (PV) resistant or intolerant to hydroxyurea (HU). An association between RUX treatment and an increased risk of SPM has been recently recognized, mainly non-melanoma skin cancers (NMSCs). Controversial data are reported regarding potential synergy and safety of ruxolitinib combination with immunotherapy (like programmed death and death-ligand, PD1/PD-L1 inhibitors) for the underlying skin cancer. We report a case of refractory PV successfully treated with ruxolitinib with a subsequent metastatic Merkel cell carcinoma (MCC). The patient received combined treatment with the anti-PD-L1 avelumab and ruxolitinib, resulting in both MPN and MCC control without safety concerns.

## Case presentation

**Figure 1:**
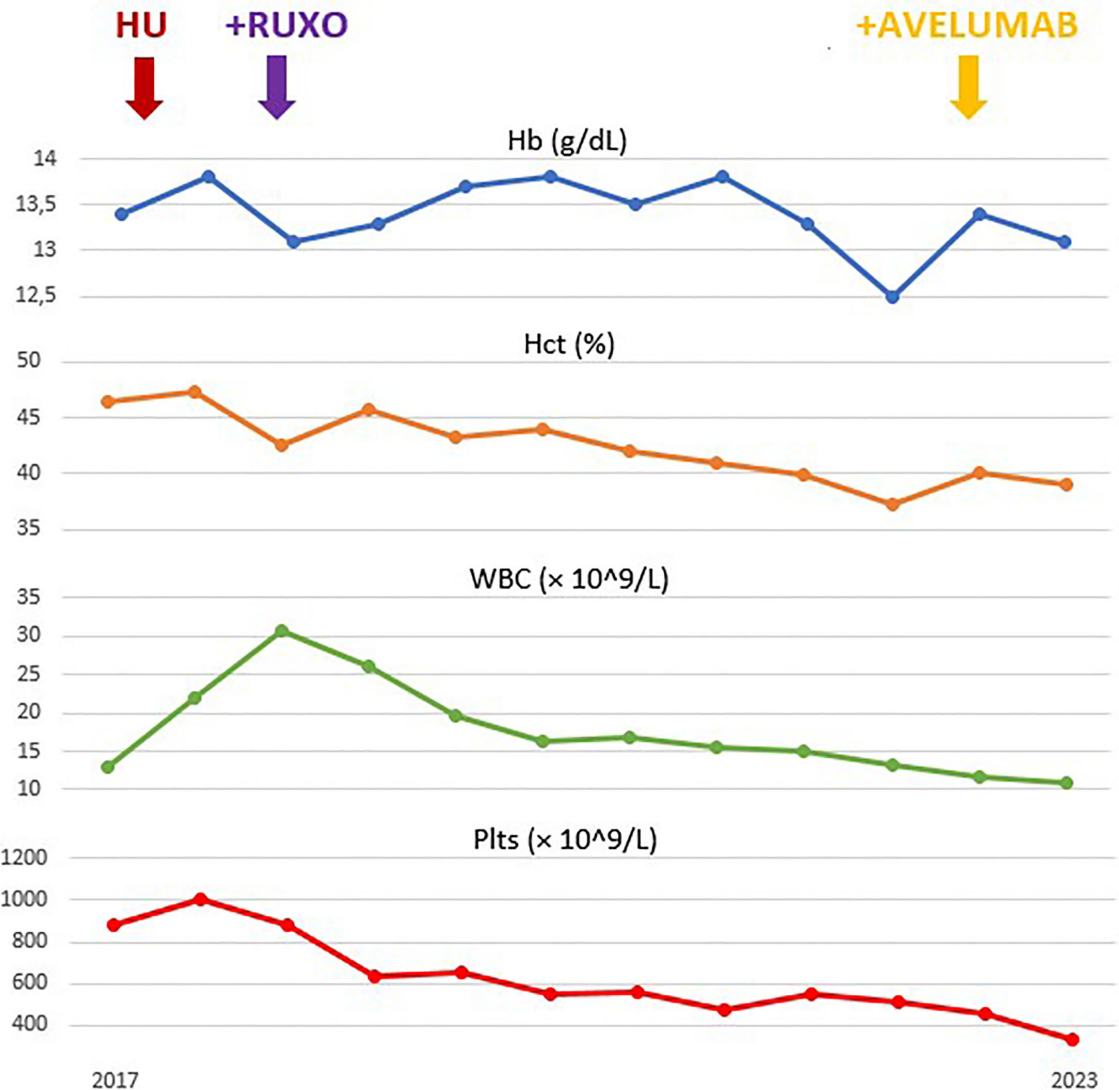
Evolution of hemoglobin (Hb), hematocrit (Hct), leukocyte (white blood cells, WBC) and platelets (Plts) count during treatment.

A 65-year-old man was diagnosed in 2014 as having a JAK2 V617F-mutated PV. According to international guidelines, he started prophylaxis with low-dose aspirin and treatment with HU. In March 2018, he developed thrombocytosis (>2.000 × 10^9^/L) and poor hematocrit control, requiring additional phlebotomies. Considering that the patient was resistant, according to European LeukemiaNet (ELN) criteria (2), he started second-line ruxolitinib at the recommended dose of 10 mg BID. After 2 months, he had complete response with hematocrit control, without requiring further phlebotomies. Soon after, ruxolitinib was reduced to 15 mg daily for grade 2 liver toxicity. In February 2019, he underwent surgery and radiotherapy for laterocervical cutaneous melanoma. In November 2021, after approximately 2 years of RUX treatment, he developed slight bilateral laterocervical swelling. In May 2022, given the evidence of neck adenopathy, left lateral cervical lymph node excision was performed, and the biopsy result was positive for metastatic high-grade neuroendocrine carcinoma. Tumor cells expressed CK20 and neuroendocrine markers and lacked TTF-1, consistent with MCC. No cutaneous MCC was identified. The staging PET/CT scan documented a Ga^68^-avid 20 mm left retroperitoneal lesion, confirming the diagnosis of metastatic Merkel cell carcinoma with unknown primary lesion (MCC-UP). At the time of MCC diagnosis, the patient was in complete hematological response, with normal blood count and without any PV-related symptoms. He started therapy with the anti-programmed cell death-ligand 1 (PD-L1) antibody avelumab, administered intravenously according to the recommended schedule of 800 mg every 2 weeks until disease progression or unacceptable toxicity. PV was still in complete response, and ruxolitinib was not withdrawn. Avelumab was started on December 2022 with a close monitoring of complete blood count and blood chemistry. In January 2023, he developed mild severe acute respiratory syndrome coronavirus 2 (SARS*-*CoV*-*2) infection and was treated with tixagevimab–cilgavimab without stopping ruxolitinib. Imaging performed after 3 months (six administrations of avelumab) to evaluate MCC revealed complete response. No hematological or extra-hematological toxicities were reported with ruxolitinib–avelumab combination in the first 5 months of follow-up (**Figure 1**). At the last evaluation, PV was in complete response according to ELN criteria (2).

## Discussion

Ruxolitinib is the only JAK1/2 inhibitor approved for HU-resistant/intolerant PV, since the pivotal trials “RESPONSE 1” and “RESPONSE 2” showed an increased rate of hematological response, reduction in splenic volume, and improvement in PV-related symptoms in comparison with best available therapy (BAT) (3, 4). However, several studies conducted in MF, such as the COMFORT-II trial, showed an increased risk of SPMs, mainly lymphoma and non-melanoma skin cancers (NMSCs) (5). Among NMSCs, Merkel cell carcinoma, a rare neuroendocrine carcinoma, is the second most common cause of skin-cancer-related death. It has been described that immune system plays an active role in the control, prevention, and progression of MCC. Approximately 10% of patients affected by MCC are on immunesuppression (6). JAK1/2 inhibitors suppress the activity of different proinflammatory cytokines and impair T lymphocytes function (7). Immune checkpoint inhibitors (ICIs) have recently demonstrated promising efficacy in metastatic MCC by enhancing the cytotoxic activity of T cells (8). There are concerns that JAK inhibitors may compromise the effectiveness of ICI in NMSC, since acquired inactivating mutations in JAK1 and JAK2 genes resulted in a reduction in PD-L1 expression in melanoma patients, leading to acquired resistance to ICI treatment (9). Conversely, JAK2 V617F mutation was also found to increase PD-L1 expression by phosphorylating STAT3/STAT5 on the surfaces of platelets, megakaryocytes, and myeloid-derived suppressor cells (MDSCs) (10). Additionally, the 9p24.1 amplification, identified in various malignancies as leading to increased PD-L1 expression, has been discovered to frequently contain focal amplification of the JAK2 gene (11). Thus, it is plausible that the PD-1/PD-L1 and JAK2 pathway could represent complementary therapeutic targets, indicating that ruxolitinib and ICI may be combined to achieve synergy. This mechanism could account for the sustained complete response in NMSCs reported in our patient and in other case reports with the combination of ICI and RUX (12, 13). In similar, limited case series, controversial data have been reported about the toxicity of combination therapy: hematological toxicity due to ruxolitinib–avelumab combination led to discontinuation of both therapies by the fourth cycle of immunotherapy in two patients (14), while a case of combination of ruxolitinib and cemiplimab without hematological concerns was also reported (15). In our patient, combined treatment was associated with the absence of hematological toxicity. As been reported previously, our case highlights the possible occurrence of solid skin cancer in patients with MPNs treated with ruxolitinib, suggesting routine dermatological screening and follow-up during continuous treatment. Considering the increasing use of JAK inhibitors, more and more patients may be diagnosed with secondary malignancies and may potentially require combination therapy. This case report offers crucial details to physicians seeking advice on how to combine these medications in the future. Combined treatment of JAK2 inhibitors with novel anti-cancer drugs is possible, after discussion of each case in multidisciplinary settings, in our case without increased toxicity and good clinical efficacy. However, further investigations are necessary to conclusively evaluate the association of the two drugs.

## Data availability statement

The original contributions presented in the study are included in the article/supplementary material. Further inquiries can be directed to the corresponding author.

## Ethics statement

Ethical review and approval was not required for the study on human participants in accordance with the local legislation and institutional requirements. Written informed consent from the patient was not required to participate in this study in accordance with the national legislation and the institutional requirements. Written informed consent was obtained from the individual(s) for the publication of any potentially identifiable images or data included in this article.

## Author contributions

CM and MP participated in the care of the patient, conceived the idea for publication, and wrote the manuscript; AC, CI, MC, IC and ES participated in the clinical care of the patient and contributed to the manuscript. MM and MB guided the direction of the report, and contributed to the manuscript. All authors contributed to the article and approved the submitted version.
